# Nitrogen Addition and Understory Removal but Not Soil Warming Increased Radial Growth of *Pinus cembra* at Treeline in the Central Austrian Alps

**DOI:** 10.3389/fpls.2018.00711

**Published:** 2018-05-29

**Authors:** Andreas Gruber, Walter Oberhuber, Gerhard Wieser

**Affiliations:** ^1^Department of Botany, University of Innsbruck, Innsbruck, Austria; ^2^Naturwerkstatt Tirol, itworks Personalservice & Beratung gemeinnützige GmbH, Zams, Austria; ^3^Department of Alpine Timberline Ecophysiology, Federal Research and Training Centre for Forests, Natural Hazards and Landscape (BFW), Innsbruck, Austria

**Keywords:** alpine treeline, *Pinus cembra*, intra- annual stem growth, soil warming, nitrogen fertilization, understory removal, competition

## Abstract

Beside low temperatures, limited tree growth at the alpine treeline may also be attributed to a lack of available soil nutrients and competition with understory vegetation. Although intra-annual stem growth of *Pinus cembra* has been studied intensively at the alpine treeline, the responses of radial growth to soil warming, soil fertilization, and below ground competition awaits clarification. In this study we quantified the effects of nitrogen (N) fertilization, soil warming, and understory removal on stem radial growth of *P. cembra* at treeline. Soil warming was achieved by roofing the forest floor with a transparent polyvinyl skin, while understory competition was prevented by shading the forest floor with a non-transparent foil around six trees each. Six trees received N- fertilization and six other trees served as controls. Stem growth was monitored with band dendrometers during the growing seasons 2012–2014. Our 3 years experiment showed that soil warming had no considerable effect on radial growth. Though understory removal through shading was accompanied by root-zone cooling, understory removal as well as N fertilization led to a significant increase in radial growth. Hardly affected was tree root biomass, while N-fertilization and understory removal significantly increased in 100-needle surface area and 100-needle dry mass, implying a higher amount of N stored in needles. Overall, our results demonstrate that beside low temperatures, tree growth at cold-climate boundaries may also be limited by root competition for nutrients between trees and understory vegetation. We conclude that tree understory interactions may also control treeline dynamics in a future changing environment.

## Introduction

Although the alpine treeline has attracted the interest of researchers for more than one century (Brockmann-Jerosch, 1919; Däniker, 1923; Wardle, 1974; Tranquillini, 1979; Körner, 1998, 2012; Holtmeier, 2003; Wieser and Tausz, 2007; Piper et al., 2016), the causes to treeline formation are still under debate. Nevertheless, it has been hypothesized that low temperatures limit growth processes in meristematic tissues (growth limitation hypothesis; Körner, 1998, 2012; Hoch and Körner, 2003). A world-wide survey across natural high elevation treelines indicates that growing season mean root-zone temperature constrains tree growth in temperature limited ecosystems (Sveinbjörnsson, 2000; Körner and Hoch, 2006). In a global survey, Körner and Paulsen (2004) found that a growing season mean soil temperature of 6.4 ± 0.7°C in 10 cm soil depths matches the upper elevational limit of tree growth. Moreover, a growth decline at the alpine treeline may also be attributed to a lack of available soil nutrients (Tranquillini, 1979; Sveinbjörnsson et al., 1992), especially nitrogen (Hoch, 2013) and competition with understory vegetation (Elliott, 2011; Grau et al., 2012; Liang et al., 2016).

Given that alpine treelines are strongly temperature limited, they are considered to be sensitive to climate warming (Walther et al., 2005; Holtmeier and Broll, 2007; Wieser et al., 2009; Liang et al., 2016; Camarero et al., 2017). So far, only a few studies evaluated the effect of soil warming on tree growth in boreal forests (Strömgren and Linder, 2002; Dao et al., 2015) and at treeline in the Swiss (Dawes et al., 2015) and French Alps (Loranger et al., 2016) with results ranging from a strong growth stimulation to no stimulation in growth. In a pilot study at treeline in the Austrian Alps, Gruber et al. (2010) investigated the effect of root-zone warming and cooling on radial growth of *Pinus cembra*. Although not statistically significant, results of this study indicated that *P. cembra* responded to soil warming with an increase and to soil cooling with a decline in stem radial growth, when compared to control trees with soil temperature left unmanipulated. Moreover, differences in stem growth with respect to soil temperature may also be attributed to varying soil nutrient contents with respect to micro topography (Anschlag et al., 2008).

Beside low temperatures, the termination of tree growth their upper distribution limit may also be attributed to a low availability of soil nutrients (Sveinbjörnsson et al., 1992) and hence also to an insufficient nutrient uptake (Susiluoto et al., 2010). Particularly total and available nitrogen (N), which is a key nutrient, is known to limit plant productivity in numerous terrestrial ecosystems (LeBauer and Treseder, 2008). This also holds for the treeline ecotone (Tranquillini, 1979) where low soil temperatures limit N mineralization and decomposition (Nadelhoffer et al., 1991; Rustad et al., 2001; Melillo et al., 2002). Yet, soil fertilization studies in boreal treelines indicated enhanced tree growth after soil fertilization (Weih, 2000; Weih and Karlsson, 2001; Susiluoto et al., 2010).

Beside the two abiotic factors, low temperature and low nutrient availability, root competition for nutrients may also have a noticeable influence on tree growth (Nilsson and Wardle, 2005; Matsushima and Chang, 2006; Elliott et al., 2015; Du et al., 2016). There are some data indicating that the absence of understory (i.e., the removal of below-ground competition) may enhance tree (Platt et al., 2004; Song et al., 2016) and seedling growth (Okano and Bert-Hane, 2015) in subalpine forests and the treeline ecotone, respectively. To our knowledge, however, the role of root competition between adult trees and understory vegetation in regulating tree growth at treeline in the Central European Alps received no attention.

Although the intra-annual stem radial increment of *P. cembra* has been studied intensively at the alpine treeline (Loris, 1981; Rossi et al., 2006, 2007; Gruber et al., 2009a,b), the responses of radial growth to soil warming, soil fertilization, and below ground competition, respectively, still awaits clarification for conifers at the alpine treeline. In this unique study, we investigated how soil temperature, N fertilization, and understory removal influences stem radial growth of *P. cembra* at treeline. Additionally, we also estimated root biomass, needle and foliar nutrient concentrations as well as specific leaf area. We hypothesized that: (1) soil warming, (2) understory removal, and (3) N fertilization will enhance radial growth of *P*. *cembra* at treeline in the Central Tyrolean Alps. Soil warming and understory removal was incited by root-zone roofing throughout three consecutive growing seasons, while continuously monitoring intra-annual changes in stem radius with electronic band dendrometers (Gruber et al., 2010; Oberhuber, 2017). In addition, some trees received a soil nitrogen fertilization. Findings are expected to contribute to an increased understanding of the importance of root competition and soil N fertilization on tree growth at treeline in the Central Austrian Alps, where mean annual air temperature increased by 0.50°C per decade, during the past 35 years (Wieser et al., 2016).

## Materials and Methods

### Study Site and Experimental Design

The study was carried out in a south exposed *P. cembra* afforestation at treeline above Haggen near St. Sigmund in the Sellrain Valley, Tyrol, Austria (“Haggener Sonnberg,” 47°12′42″N, 11°05′04″E, 2150 m a.s.l.). Slope angel and aspect were 25° and SSW, respectively (Kronfuss, 1997). The long term mean annual temperature (1975–1994) at a nearby weather station at 1800 m a.s.l. (Kronfuss, 1997) was 3.2°C and the mean annual precipitation was 909 mm. For the same period the growing season (May through September) had a mean air temperature of 8.5°C and a mean precipitation of 537 mm. The soil at our study site is a podzolic cambisol (Neuwinger, 1972; World Base for soil Resources classification, FAO, 2008) which derived from gneisses and mica schist bedrock (Kronfuss and Havranek, 1999). The sandy loam subsoil was covered by a ca. 5 cm thick humus layer (Wieser et al., 2015). Hydraulic field capacity at -0.033 MPa (sensu Blume et al., 2010) of the top sub soil (0–25 cm) is 25% volume and the top soil is enriched by 8% of organic matter (Wieser et al., 2015).

The stand formed a sparsely open canopy permitting a dense understory of herbaceous species together with some dwarf shrubs. During the study period (2011–2014) the trees were ≈25 years old, with a stem diameter at breast height of 7.1 ± 1.2 cm, and an average height of 3.4 ± 0.4 m. Trees selected for the experiment were separated at least by a distance of 3–5 m. In early summer 2011 we established 24 quadratic 16 m^2^ plots with one *P. cembra* tree in the center, resulting in six plots each of the four experimental groups: (1) nitrogen (N) fertilization (N treatment), (2) an increase in soil temperature (warming treatment), (3) elimination of understory vegetation (understory removal treatment), and (4) controls, for with soil temperature left un-manipulated, the understory vegetation was present (competition), and trees were not fertilized (control treatment). In order to facilitate the logistics of soil temperature and soil moisture monitoring (see below), three adjacent plots per treatment were assembled into a block.

The nitrogen plots were fertilized following the recommendations of Kilian and Müller (1994). The plots were fertilized twice, in spring 2012 and 2013, with 10 g m^-2^ calcium ammonium nitrate (NAC 27 N; Borealis L.A.T., AT) containing 27% nitrogen with equal parts of NO_3_-N and NH_4_ and 12% CaO; the latter counteracting potential effects of N acidification. Soil warming was achieved by roofing the forest floor with a heat trapping 0.5-mm thick transparent polyvinyl skin (Wieser et al., 2015). In the no understory plots, herbaceous species and dwarf shrubs were completely removed in July 2011. This condition was maintained by shading the forest floor with a non-transparent foil. The respective foils were fixed on frames 15 cm above ground. The sides were open for allowing air circulation. Surface and slope run-off of water was allowed to penetrate the soil during and after rainfall (in total 67% of precipitation; Neuwinger et al., 1988; Wieser et al., 2015). Soil warming and understory removal through shading operated from end July throughout October 2011, and continued during the snow-free periods (May–October) of 2012, 2013, and 2014.

As expected, understory removal through shading was accompanied by root-zone cooling. Additionally, there is also evidence that artificial soil cooling by roofing the forest floor may have negative effects on stem radial growth of *P. cembra* growing in the *krummholz* (crippled trees) limit at 2180 m a.s.l. on Mt. Patscherkofel south of Innsbruck, Austria (Gruber et al., 2010). Therefore, in order to discriminate between potential antagonistic effects of understory removal and soil cooling on radial growth, we also removed the understory in all the control and warmed plots in early April 2014 and covered the ground with a permeable weed fleece to avoid any re-growth of the understory vegetation throughout the entire growing season of 2014. Detailed information on potential understory competition with respect to treatment is given in **Table 1**.

**Table 1:** Understory characteristics in the control, the warming, the understory removal, and the N fertilization treatment during the growing seasons 2012, 2013, and 2014.

Treatment	2012	2013	2014
Control	Intact understory	Intact understory	No understory
Warming	Intact understory	Intact understory	No understory
Understory removal	No understory	No understory	No understory
N treatment	Intact understory	Intact understory	

### Environmental Measurements and Dendrometer Records

Air temperature (*T*_air_) and relative humidity (CS215 Temperature and Relative Humidity Sensor), solar radiation (SP1110 Pyranometer Sensor), wind velocity (A100R Anemometer) and precipitation (ARG100 Rain Gauge, all sensors Campbell Scientific, Shepshed, United Kingdom) were measured continuously at 2 m height at the study site. In order to examine differences in the seasonal course of soil temperature (*T*_soil_) and volumetric soil water content (𝜃) between control, warming, and understory removal blocks, six soil temperature probes (T 107 Temperature Probe, Campbell Scientific, Shepshed, United Kingdom) and two soil moisture sensors (EC 5 Soil Moisture Sensor, Decagon Devices Inc. Pullman, WA, United States) were installed in each block close to the trees used for dendrometer records. While 𝜃 was measured at 10 cm soil depth solely, *T*_soil_ was measured at 5, 10, and 20 cm soil depth (two probes per depth and block). To evaluate a potential influence of forest-floor roofing on stem temperature (*T*_stem_), a T 107 Temperature Probe (Campbell Scientific, Shepshed, United Kingdom) was mounted at 50 cm stem height on the north facing side of all the trees in control, warmed and understory removal treatment, respectively. All the environmental data were recorded with two CR1000 data loggers (Campbell Scientific, Shepshed, United Kingdom) programmed to record 30-min averages of measurements taken every minute.

Intra-annual changes in stem radius of all the selected study trees were continually monitored using electronic band dendrometers with automatic temperature compensation (DC2, Ecomatik, Dachau, Germany) installed 0.5 m aboveground in early May 2011 to record intra-annual radial growth throughout the snow free periods (April–October) of 2012, 2013, and 2014. The measuring cable consisted of Invar-steel with a thermal expansion coefficient <1.4 × 10^-6^ K. Dead outermost layers (periderm) of the bark were slightly removed to reduce the influence of hygroscopic swelling and shrinkage of the bark on dendrometer traces and to ensure close contact with the stem (cf. Gruber et al., 2009a,b; Oberhuber, 2017). Data were recorded with a CR1000 data logger (Campbell Scientific, Shepshed, United Kingdom) programmed to record 30-min averages of measurements taken every minute. Circumference variations derived from band dendrometers were transformed to radial variations, and daily stem radius variations were determined by calculating the difference between mean values of two consecutive days (“daily mean approach,” Deslauriers et al., 2007). As for dendrometers no unambiguous date of growth onset can be determined (Downes et al., 1999; Deslauriers et al., 2003; Gruber et al., 2009b), we defined the onset of radial growth as the date when radial increment permanently exceeded the initial value after snow melt in May. To separate daily patterns of water movement from irreversible expansion growth, we applied a Gompertz function for describing the long-term development of radial growth over an entire growing season according to Rossi et al. (2006):

$$\begin{array}{c} y\;=\;A\;*\exp[-\exp(\beta - \kappa t)] \end{array}$$

where *y* is the cumulative sum of growth, *A* is the upper asymptote of the total annual radial growth, β is the *x*-axis placement parameter, κ is the rate of change parameter, and t is the time computed in Julian days. The inflection point (*I*_p_) corresponding to the maximum value of the radial growth rate was calculated as *I*_p_ = β/κ (Rossi et al., 2006). Finally, as dendrometer records are affected by stem water status, the end of stem radial growth was considered when 90% of *A* given by the Gompertz function was reached (Gruber et al., 2009b).

### Additional Measurements

Tree characteristics including specific leaf area (SLA), 100 needle dry weight, and foliar nutrient concentrations of all the study trees, as well as root biomass at the plot level were determined in late fall 2013 (November 18, 2013). SLA (cm^2^ g^-1^) was calculated from measured needle dry weight and measured projected needle surface area. For nutrient analyses the needles were dried to constant weight at 60°C, ground and stored dry before analysis. The concentrations of nitrogen, phosphor and potassium were estimated according to EPA 3052^1^. For assessing root biomass mass (including tree and understory vegetation roots) at the plot level we sampled six soil cores (volume 125 cm^3^) per treatment in 0–10 and in 10–20 cm soil depth, respectively. Roots were rinsed with tap water in a sieving cascade to remove all soil particles while minimizing the loss of fine roots. Subsequently, the roots were divided into fine (diameter ≤ 1 mm) and coarse roots (diameter > 1 mm) and dried to constant weight at 75°C before determining dry weight. Finally, increment cores (5 mm in diameter) were taken at sensor height in fall 2014 for estimating radial increment in 2010, the year preceding the experiment.

### Data Analysis

As daily mean *T*_soil_ and 𝜃 values in the two control, the two warmed, and the two understory removal blocks, as well as *T*_stem_ of all the trees under study did not differ significantly [all *P*-values > 0.1; one-way analysis of variance (ANOVA)], we used repeated-measures ANOVA to test for differences in *T*_soil_, 𝜃, and *T*_stem_ between control, warmed, and understory removal blocks. Differences in the overall mean parameters of the Gompertz functions for modeling intra-annual radial growth (upper asymptote, inflection point, rate of change parameter, time when 90% of increment were produced) between the control, the warming, the understory removal, and the fertilization treatment during the growing seasons 2012, 2013, and 2014 were tested for significance using analysis of variance (ANOVA) and multiple comparison Tukey’s HSD tests.

We used binary logistic regressions (logit models; SPSS for Windows) to determine the probability of radial growth being active at a given air and soil temperature. Values of daily radial increment where binary coded as: no increment (value 0) or increment (value 1) during the period May 1–September 30. The model was fitted for each tree, treatment and year with the respective temperature series; i.e., daily mean air and soil temperature in 10 cm soil depth. Temperature thresholds were calculated when the probability of radial increment was 0.5 (cf. Rossi et al., 2007). Fitting verification included χ^2^ of the likelihood ratio, Wald’s χ^2^ for regression parameter and goodness of fit, and Hosmer–Lemeshow Ĉ for eventual lack of fit. Finally, thermal thresholds were averaged over the three snow free periods 2012, 2013, and 2014 and compared between treatments (control, warmed, understory removal ≈ cooling) using ANOVA.

ANOVA was also used for testing differences in SLA, needle area, needle mass, needle nutrient concentrations, and total fine root mass between treatments. A probability level of *P* < 0.05 was considered as statistically significant and *P*-values ≥ 0.05 and < 0.1 as marginally significant. Statistical analyses were made with the IBM SPSS Statistics 21 (IBM, NY, United States).

## Results

### Environmental Conditions

**FIGURE 1:**
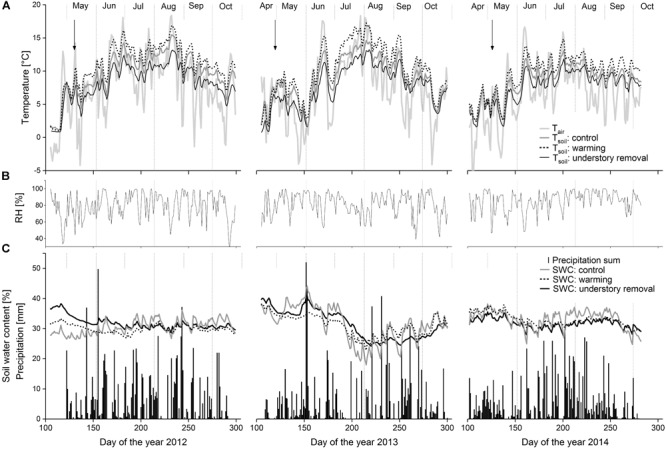
**(A)** Seasonal course of daily mean air temperature and daily mean soil temperatures in 10 cm soil depth in control, warming and understory removal blocks. **(B)** Relative air humidity. **(C)** Daily sum of precipitation and daily mean soil water content in 10 cm soil depth in control, warming and understory removal blocks from April to October 2012, 2013, and 2014. Arrows indicate roof closure.

Growing season (May–September) mean *T*_air_ (**Figure 1**) was 9.0°C in 2012, 8.1°C in 2013, and 7.3°C in 2014. Daily mean relative humidity varied between 50% during cloudless warm days and close to 100% on overcast rainy days (**Figure 1**). Stem temperature at 50 cm stem height was not considerably altered by the experimental setup (data not shown; *P-*values from repeated measure analysis: *P* = 0.215, *P* = 0.418, and *P* = 0.390 for 2012, 2013, and 2014, respectively).

Independent of treatment, *T*_soil_ generally followed seasonal trends in *T*_air_ (**Figure 1**). Warming caused *T*_soil_ to average +1.5 ± 0.5, +1.3 ± 0.2, and +1.0 ± 0.2°C above the corresponding control levels at 5, 10, and 20 cm soil depth, respectively (all *P* < 0.05; **Table 2**). In the understory removal blocks by contrast, shading caused *T*_soil_ to be at average -3.0 ± 0.8, -2.6 ± 0.5, and -2.4 ± 0.4°C below the corresponding control levels at 5, 10, and 20 cm soil depth, respectively (all *P* < 0.05; **Table 1**).

**Table 2:** Seasonal average soil temperature (*T*_soil_; °C) at 5, 10, and 20 cm soil depth and volumetric soil water content (𝜃) at 10 cm soil depth, in control, warmed and understory removal blocks for the periods May 1– September 30, 2012, 2013, and 2014.

Year	Treatment	*T*_soil_ 5 cm	*T*_soil_ 10 cm	*T*_soil_ 20 cm	𝜃 10 cm
2012	Control	11.2 ± 0.2^a^	10.7 ± 0.2^a^	10.0 ± 0.2^a^	30.5 ± 0.2^a^
	Warming	12.4 ± 0.2^b^	11.9 ± 0.2^b^	11.1 ± 0.2^b^	30.0 ± 0.1^a^
	Understory removal	9.1 ± 0.2^c^	9.1 ± 0.2^c^	8.4 ± 0.2^c^	30.5 ± 0.2^a^
2013	Control	10.1 ± 0.3^a^	9.8 ± 0.3^a^	9.4 ± 0.2^a^	31.0 ± 0.2^a^
	Warming	12.1 ± 0.3^b^	11.3 ± 0.3^b^	10.5 ± 0.3^b^	30.5 ± 0.3^a^
	Understory removal	8.6 ± 0.3^c^	8.3 ± 0.2^c^	7.9 ± 0.2^c^	28.3 ± 0.4^a^
2014	Control	9.3 ± 0.2^a^	9.1 ± 0.2^a^	8.9 ± 0.2^a^	31.0 ± 0.2^a^
	Warming	10.5 ± 0.2^b^	10.2 ± 0.2^b^	9.7 ± 0.2^b^	32.9 ± 0.1^a^
	Understory removal	8.4 ± 0.2^c^	8.1 ± 0.2^c^	7.8 ± 0.2^c^	31.6 ± 0.1^a^

*Values are the mean ± SE of four sensors per treatment. Different letters indicate significant differences at p < 0.05.*

Precipitation during the growing seasons 2012, 2013, and 2014 amounted 940, 806, and 785 mm, respectively. Due to frequent precipitation over the entire growing seasons (**Figure 1**) soil water content (𝜃) in 10 cm soil varied between 44 and 19% vol. (**Figure 1**). Average 𝜃 over the growing seasons 2012, 2013, and 2014 did not differ significantly (all *P* > 0.64) between control (30.8 ± 0.3% vol.), warmed (31.1 ± 1.6% vol.) and understory removal (shaded) blocks (30.1 ± 1.7% vol.), respectively (**Table 2**).

### Treatment Effects on Growth

**FIGURE 2:**
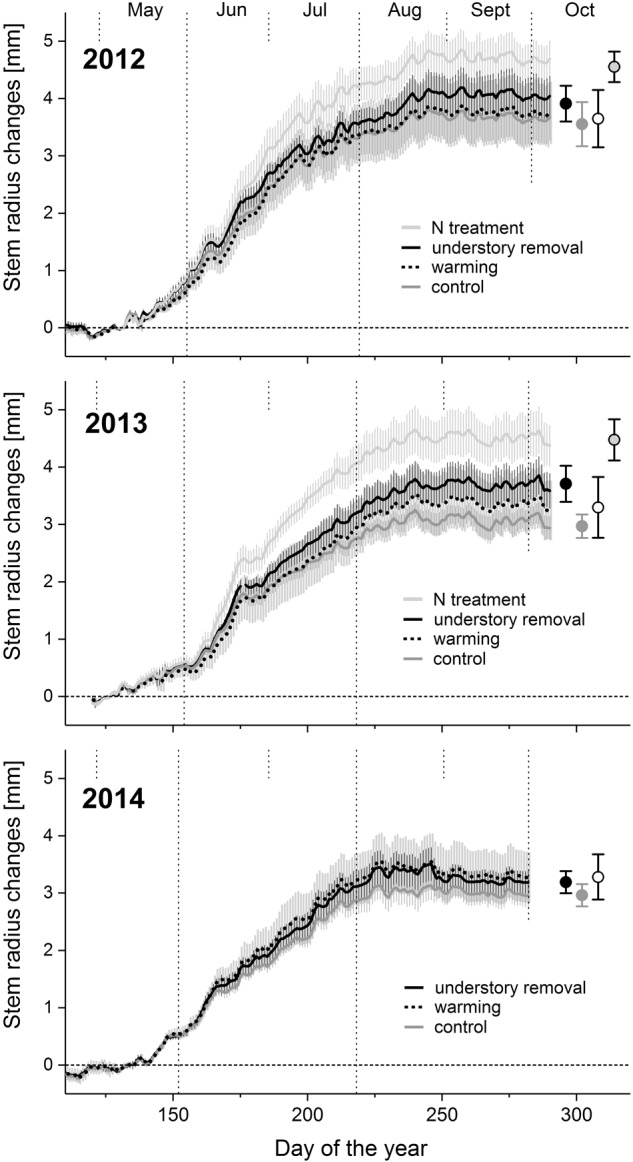
Time series of mean daily *Pinus cembra* dendrometer records and standard deviations of the control, warming, understory removal and the N during growing seasons 2012, 2013, and 2014. Circles denote mean upper asymptotes ± SD of the Gompertz functions of each treatment.

In 2010, the year preceding the experiment, annual radial stem increment did not differ significantly (all *P* > 0.45) between the treatments and averaged 3.5 ± 0.7, 3.4 ± 0.5, 3.9 ± 0.5, and 3.8 ± 0.7 mm in the control, the warming, the understory removal, and the fertilization treatment, respectively. The corresponding values for 2011 were 3.2 ± 0.7, 3.1 ± 1.0, 3.5 ± 0.4, and 3.4 ± 0.6 mm, respectively. Independent of treatment and year, radial growth started in the 2nd week of May (doy 128–133; **Figure 2**). Based on the calculated Gompertz functions, maximum daily radial growth peaked in June around summer solstice (doy 167–173) and radial growth ended in early August (doy 218–227), with no treatment effects (**Table 3**). Treatment, however, affected total annual stem radial increment (**Table 3**), which corresponds to the upper asymptote of the Gompertz function (**Figure 2**). Fertilization caused annual radial growth to increase significantly by 28 and 48% in 2012 and 2013, respectively, (both *P* < 0.004) above the level of the control treatment (**Figure 2** and **Table 3**). Understory removal caused radial growth to increase marginally significant on average by 11% (*P* = 0.08) in 2012, and significantly on average by 22% (*P* = 0.04) in 2013 above the level of control trees (**Figure 2** and **Table 3**). However, it has to be noted that understory removal through shading was accompanied by significant cooling of the rooting-zone (cf. **Figure 1** and **Table 2**). Warmed and control trees by contrast, resembled each other with respect to radial growth (**Figure 2**), in the absence of a warming effect on annual stem radial increment in 2012 and 2013 (**Table 3**). Removing the understory in 2014 in all the control and warmed plots also failed to find significant temperature effects (both *P* > 0.35) on total annual stem radial increment between the control, the warming and the understory removal treatment (**Table 3**).

**Table 3:** Parameters of the Gompertz function (Mean ± SD) for intra-annual radial growth of *Pinus cembra* trees selected for dendrometer records in 2012, 2013, and 2014.

Year	Treatment	*I*_p_ (DOY)	κ	90% DOY	*A* (mm)
2012	Control	167 ± 0.3^a^	0.044 ± 0.003^a^	218 ± 3.5^a^	3.71 ± 0.41^a^
	Warming	168 ± 1.9^a^	0.043 ± 0.003^a^	221 ± 5.0^a^	3.80 ± 0.54^a^
	Understory removal	166 ± 2.4^a^	0.041 ± 0.005^a^	222 ± 8.0^a^	4.11 ± 0.37^a∗^
	N treatment	168 ± 2.2^a^	0.043 ± 0.003^a^	220 ± 4.8^a^	4.74 ± 0.31^b^
2013	Control	168 ± 3.0^a^	0.043 ± 0.003^a^	221 ± 5.6^a^	3.09 ± 0.20^a^
	Warming	173 ± 3.6^a^	0.041 ± 0.002^a^	227 ± 3.9^a^	3.49 ± 0.60^ab^
	Understory removal	171 ± 2.6^a^	0.041 ± 0.003^a^	223 ± 8.4^a^	3.83 ± 0.34^b^
	N treatment	171 ± 2.6^a^	0.044 ± 0.002^a^	222 ± 3.8^a^	4.59 ± 0.43^c^
2014	Control	167 ± 1.3^a^	0.042 ± 0.001^a^	220 ± 1.7^a^	3.08 ± 0.53^a^
	Warming	167 ± 2.6^a^	0.044 ± 0.003^a^	219 ± 6.0^a^	3.48 ± 0.46^a^
	Understory removal	169 ± 2.5^a^	0.040 ± 0.003^a^	227 ± 7.3^a^	3.54 ± 0.45^a^

*Different letters indicate significant differences at P < 0.05, ^∗^ marginally significant different to control at P = 0.08. I_p_, inflection point; DOY, day of the year; 90% DOY, time when 90% of increment was produced; κ, rate of change parameter, A upper asymptote. Values are the mean ± SD of six trees per treatment.*

### Threshold Temperatures

The calculated threshold temperatures at which radial growth had a 0.5 probability of being active were averaged over the three growing seasons 2012, 2013, and 2014. Irrespective of treatment, the mean air temperature at which radial growth of *P. cembra* was active in control, warmed and understory removal plots varied between 5.1 and 5.9°C (**Table 4**; all *P* > 0.05). The mean soil temperatures at which there was a 0.5 probability of radial growth were in general higher than the air temperature thresholds, being 7.8 ± 0.4, 9.1 ± 0.6 and 6.0 ± 0.6°C in the control, the warmed, and the understory removal treatment, respectively (**Table 4**; all *P* < 0.05).

**Table 4:** Mean threshold temperatures corresponding with the 0.5-probability of active radial growth of *Pinus cembra* in the control, the warmed and the understory removal (≈cooling) treatment estimated during the growing seasons 2012, 2013, and 2014.

Treatment	*T*_air_ (°C)	*T*_soil_ (°C)
Control	5.9 ± 0.6^a^	7.8 ± 0.4^a^
Warmed	5.9 ± 0.8^a^	9.1 ± 0.6^b^
Understory removal	5.1 ± 0.9^a^	6.0 ± 0.6^c^

*Different letters indicate significant differences at P < 0.05.*

### Specific Leaf Area, Foliar Nutrient Concentrations of the Study Trees and Plot-Level Fine Root Biomass

**FIGURE 3:**
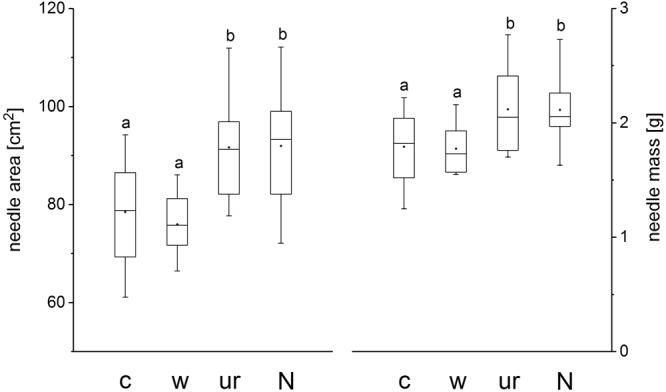
Box plots showing the median, lower (25%) and upper (75%) quartile and minimum and maximum of 100 needle area and 100 needle mass of *Pinus cembra* trees in control (c), warmed (w), understory removal (ur) and N fertilized (N) plots at the end of the growing season 2013. Different letters indicate significant differences at *P* < 0.05.

Treatment did not influence SLA (*P* = 0.98). At the end of the growing season 2013 current-year needle SLA averaged 43.5 ± 1.4 cm^2^ g^-1^ across all treatments. Current year 100-needle surface area and the corresponding 100-needle dry weight, however, were significantly higher in the understory removal and fertilization treatment than in the warming and the control treatment (**Figure 3**). Treatment had no effect in foliar nutrient concentrations. Averaged across all the four treatments nitrogen, phosphor and potassium concentrations of current-year needles were 18.3 ± 1.3, 1.9 ± 01, and 5.7 ± 0.4 mg g^-1^, respectively, at the end of the growing season 2013.

The impact of understory removal is also reflected at the plot level. After three growing seasons total fine-root biomass mass (including tree and understory vegetation roots) in 0–10 cm soil depth was significantly lower in understory removal plots as compared to control and warmed (both *P* < 0.05) plots (**Table 5**). Although statistically not significant, total fine-root biomass mass in fertilized plots was considerably reduced when compared to warmed, and control plots, but considerably higher than in understory removal plots (**Table 5**). Treatment, however, had no effect on tree root biomass in 0–10 cm soil depth as well as on total fine-root biomass in the 10–20 cm soil layer (**Table 5**).

**Table 5:** Total (tree and understory) and tree fine-root biomass (g _dw_ l^-1^ soil); (Mean ± SD) in 0–10 cm and 10–20 cm soil depth in control (c), warmed (w), understory removal (ur) and N fertilized (N) blocks at the end of the growing season 2013.

Soil depth	Root biomass	c	w	ur	N t
0–10 cm	Tree and understory^1^	9.4 ± 2.9^a^	9.8 ± 2.0^a^	4.5 ± 1.6^b^	7.6 ± 1.4^ab^
0–10 cm	Tree^2^	1.0 ± 0.9^a^	1.2 ± 1.6^a^	0.8 ± 0.6^a^	0.7 ± 0.7^a^
10–20 cm	Tree and understory^1^	3.1 ± 1.4^a^	3.5 ± 0.6^a^	3.0 ± 1.1^a^	3.1 ± 0.7^a^

*^*1*^This study. ^*2*^Data from Rainer et al. (2015). Different letters indicate significant differences at P < 0.05.*

## Discussion

In this study, we tested the hypothesis that stem radial growth of *P. cembra* at treeline in the Central Tyrolean Alps is enhanced by soil warming, understory removal, as well as N-fertilization. Our experimental approach was appropriate to manipulate *T*_soil_, competition and N availability, enabling the clarification of radial growth *in situ* under a wide range of conditions. Although the roofing prevented 33% of growing season precipitation to reach the soil, treatment differences in daily mean 𝜃 between control, warmed and understory removal blocks stayed within the typical variation of at the study site (Neuwinger, 1972) which confirmed that the employed roofing system did not prevent any shortage in soil water availability (**Figure 1** and **Table 2**). This is further corroborated by sap flow measurements, where roofing caused sap flow density of *P. cembra* to increase above the levels of control trees, whereas leaf level net CO_2_ uptake, conductance for water vapor, and water-use efficiency stayed unchanged (Wieser et al., 2015). Therefore, we assumed that our roofing induced 33% reduction of growing season precipitation reaching the soil did not considerably influence radial growth as a potential “side-effect” due to soil roofing.

After 3 years of treatment, our results indicate that soil warming had no considerable effect on radial growth, which allows hypothesis (1) to be rejected. Although understory removal through shading was accompanied by root-zone cooling (**Figure 1** and **Table 2**), understory removal as well as soil N-fertilization significantly increased radial growth, confirming hypothesis (2) and (3). Despite the fact that growing season stem radial growth of trees in the understory removal and the N addition treatment was significantly larger than in the control and warming treatment (**Figure 2** and **Table 3**), we observed no considerable differences in the dynamics of tree growth (**Figure 2**).

Similar to our findings, Gruber et al. (2010) also failed to find a significant increase in radial growth of *P. cembra* in the *kampfzone*, i.e., the upper border line of the treeline ecotone (Körner, 2012), on Mt. Patscherkofel south of Innsbruck (2.180 m a.s.l.) after 1 year of 0.5°C soil warming. An increase in soil temperature of 4°C and as a consequence a 1-week earlier snow melt also did not affect growth and cambial phenology of *Picea mariana* in a boreal forest in Canada (Lupi et al., 2012). Dao et al. (2015) also failed to find substantial effects on xylem phenology and cell production of mature black spruce after 6 years of soil warming by 4°C in a boreal forest. As in our study, 6 years of soil warming by 3.2°C in 10 cm soil depth at treeline in the Swiss Alps (2.180 m a.s.l.) also resulted in no considerable increase in the growth of *P. uncinata*, and *Larix decidua* during the first 2 years of the experiment (Hagedorn et al., 2010), while in the following 4 years slightly improved shoot growth persisted in *P. uncinata*, but diminished in *L. decidua* (Dawes et al., 2017). These observed differences in the response to soil warming may be attributed to differences in the rooting depth between pine and larch, because coarse roots of larch can penetrate into deeper soil layers when compared to pine (Kutschera and Lichtenegger, 2002), and thus may probably have not experienced the warming treatment (Dawes et al., 2015). Strömgren and Linder (2002) reported an increase in stem production of boreal *Picea abies* trees in a 7-year study combining soil warming and nutrition.

The absence of significant growth stimulation due to soil warming in our study suggests that growth stimulation due to increasing soil temperatures may not be expected for *P. cembra* trees at treeline in the Central European Alps in the future. However, a potential effect of soil warming may occur under a longer period of experimentation (Dao et al., 2015), as there is evidence that *P. cembra* exhibits determinate shoot growth, in any year growth is primarily impelled by the bud formation occurring in the previous year (Tranquillini, 1979). Additionally, in alpine ecosystems, growth response to warming is mediated through a time lag by one or more years after initiation of warming (Danby and Hik, 2007; Hagedorn et al., 2010). Responses during the extraordinary warm year of 2003 support our result, as the growth of *P. cembra* at the treeline in the Austrian Alps was hardly affected by a 4°C warmer summer (Oberhuber et al., 2008).

As understory removal by shading was accompanied by a significant cooling of the rooting-zone (**Figure 1** and **Table 2**), we were able to calculate threshold temperatures above which significant tree growth occurs (for a review see Körner, 2006). Körner and Paulsen (2004) found that a mean annual air temperature and 10 cm soil temperature threshold of 5.6–8.5°C and 6.4°C, respectively, define the upper elevational limit of tree growth worldwide. For *P. cembra* in the Italian Alps, Rossi et al. (2007) estimated a daily threshold mean air temperature when radial growth was active of 5.8–8.6°C. In our 3-year study period radial growth was active when mean air temperature was above 5.1–5.9°C (**Table 4**). Hence, our results support the existence of a comparable air temperature threshold for radial growth in *P. cembra* at the alpine treeline. Soil temperature thresholds by contrast differed significantly between treatments and ranged from 6.0°C in trees experiencing understory removal (≈cooling) to 9.1°C in trees experiencing soil warming (**Table 4**), indicating that soil temperature is not the main limiting factor for radial growth for *P. cembra* at treeline (Rossi et al., 2007; Lupi et al., 2012).

Beside low temperatures, low soil nutrient availability may also affect tree growth at high altitude and latitude (Tranquillini, 1979; Jarvis and Linder, 2000; Rossi et al., 2011) because low soil temperatures limit mineralization, decomposition, and N-cycling (Nadelhoffer et al., 1991; Rustad et al., 2001; Melillo et al., 2002). Thus, on the long term future climate warming may affect wood production, although no short term effect could be observed (Vaganov et al., 1999; Jarvis and Linder, 2000; Rustad et al., 2001; Rossi et al., 2007). Additionally, there is also evidence that fertilization effects may increase under conditions of experimental soil warming as shown for seedlings (Weih, 2000; Hoch, 2013) and mature forest trees (Rustad et al., 2001; Melillo et al., 2011; Lupi et al., 2012; but see Dao et al., 2015). In their soil heating study, Strömgren and Linder (2002) attributed the observed growth stimulation to an increase in N mineralization. The enhanced growth of *P. uncinata* at treeline in the Swiss Alps during the first 3 years of soil warming has also been attributed to a warming induced increase in soil N mineralization (Dawes et al., 2017). However, this was not investigated in our study.

As shown previously for boreal treeline sites (Sveinbjörnsson et al., 1992; Weih, 2000; Weih and Karlsson, 2001; Susiluoto et al., 2010), N-fertilization significantly increased radial growth of *P. cembra* at our study site (**Figure 2** and **Table 3**). Similar to N-fertilization, understory removal, and thus removal of root competition, also significantly enhanced stem growth of *P. cembra* (**Figure 2** and **Table 3**). Removal of below-ground competition also enhanced tree (Platt et al., 2004; Song et al., 2016) and seedling growth (Okano and Bert-Hane, 2015) in subalpine forests and in the treeline ecotone, respectively. In addition, there is also evidence that the existence of understory can limit seedling and tree establishment above the current treeline (Smith et al., 2009; Batllori et al., 2010; Elliott, 2011; Grau et al., 2012). Assessment of long term changes in species interaction, however, are still a matter of debate (Matsushima and Chang, 2006; Liang et al., 2016; Camarero et al., 2017), and await clarification for the Central European Alps.

Nevertheless, beside enhanced stem growth, N-fertilization, as well as understory removal led to a significant increase in 100-needle surface area and 100-needle dry mass (**Figure 3**) which may be attributed to a considerable increase in N uptake, although needle N concentration did not vary between treatments. Because a greater needle size with equal N concentrations implies a higher amount of N stored in needles, N uptake seemed indeed to be a limiting factor at our study site as trees did profit from N addition and understory removal both, at the stem- and the canopy level. Moreover, at the plot level, understory removal significantly reduced total (=tree and understory vegetation) fine-root biomass in 0–10 cm soil depth, while tree root biomass was hardly affected when compared to control plots (**Table 5**). Thus our findings suggest that at treeline in the Central Austrian Alps root competition for nutrients as well as low soil nutrient availability, rather than soil warming limits tree growth of *P. cembra*.

## Conclusion

Results of this study on the effect of soil warming, N fertilization, and understory removal on stem radial growth of *P. cembra* at treeline clearly showed that warming failed to induce enhanced stem growth, which allowed hypothesis (1) to be rejected. Understory removal as well as soil N-fertilization by contrast, significantly increased radial growth, confirming hypothesis (2) and (3). As hypothesis (1) was rejected, results of this study strongly suggest that beside low air temperatures, the termination of tree growth at cold-climate tree life boundaries may also be attributed to root competition for nutrients between trees and understory vegetation (Nilsson and Wardle, 2005; Matsushima and Chang, 2006; Elliott et al., 2015; Du et al., 2016). Finally, the presented results underscore the importance of including both temperature manipulation and species interactions in future studies on tree growth within the treeline ecotone.

## Author Contributions

AG, WO, and GW conceived and designed the experiments and analyzed the data. AG performed the experiments. GW, wrote the manuscript. WO and AG provided editorial advice.

## Conflict of Interest Statement

The authors declare that the research was conducted in the absence of any commercial or financial relationships that could be construed as a potential conflict of interest.

## Footnotes

1 https://www.epa.gov/

## References

[B1] Anschlag K. Broll G. Holtmeier F.-K. (2008). Mountain birch seedlings in the treeline ecotone, subarctic Finland: variation in above- and below-ground growth depending on microtopography. *Arctic Antarct. Alp. Res.* 40 609–616. 10.1657/1523-0430(07-087)[ANSCHLAG]2.0.CO;2

[B2] Batllori E. Camarero J. J. Gutierrez E. (2010). Current regeneration patterns at the treeline in the Pyrenees indicate similar recruitment processes irrespective to the past disturbance regime. *J. Biogeogr.* 37 1938–1950.

[B3] Blume H. P. Brümmer G. W. Horn R. Kandeler E. Kögl-Knabler I. Kretzschmar R. (2010). *Scheffer/Schachtchabel: Lehrbuch der Bodenkunde.* Heidelberg: Spektrum Akademischer Verlag.

[B4] Brockmann-Jerosch H. (1919). *Baumgrenze und Klimacharakter. Pflanzengeographische Kommission der Schweizerischen Naturforschenden Gesellschaft. Beiträge zur Landesaufnahme 6.* Zürich: Rascher und Cie.

[B5] Camarero J. J. Linares J. C. Garcia-Cervigon A. I. Batllori E. Martinez I. Gutierrez E. (2017). Back to the future: the responses of alpine treelines to climate warming are constrained by the current ecotone structure. *Ecosystems* 20 683–700. 10.1007/s10021-016-0046-3

[B6] Danby R. K. Hik D. S. (2007). Responses of white spruce (*Picea glauca*) to experimental warming at a subarctic alpine treeline. *Glob. Change Biol.* 13 437–451. 10.1186/s40064-015-0833-x 25729635 PMC4339320

[B7] Däniker A. (1923). Biologische Studien über Baum- und Waldgrenzen, insbesondere über die klimatischen Ursachen und deren Zusammenhänge. *Vierteljahresschrift Naturforsch. Ges. Zürich* 68 1–102.

[B8] Dao M. C. E. Rossi R. Walsh D. Morin H. Houle D. (2015). A 6-yer-long manipulation with soil warming and canopy nitrogen additions does not affect xylem phenology and cell production of mature black spruce. *Front. Plant Sci.* 6:877. 10.3389/fpls.2015.00877 26617610 PMC4643123

[B9] Dawes M. A. Philipson C. D. Fonti P. Bebi P. Hättenschwiler S. Hagedorn F. (2015). Soil warming and CO_2_ enrichment induce biomass shifts in alpüine treeline vegetation. *Glob. Change Biol.* 21 2005–2021. 10.1111/gcb.12819 25471674

[B10] Dawes M. A. Schleppi P. Hättenschwiler P. Hagedorn F. (2017). Soil warming opens the nitrogen cycle at the alpine treeline. *Glob. Change Biol.* 23 421–434. 10.1111/gcb.13365 27207568

[B11] Deslauriers A. Anfodillo T. Rossi S. Carraro V. (2007). Using simple causal modelling to understand how water and temperature affect daily stem radial variation in trees. *Tree Physiol.* 27 1125–1136. 10.1093/treephys/27.8.1125 17472939

[B12] Deslauriers A. Morin H. Begin Y. (2003). Cellular phenology of annual ring formation of *Abies balsamea* in the Quebec boreal forest (Canada). *Can. J. For. Res.* 33 190–200. 10.1093/treephys/tpx082 28985379

[B13] Downes G. Beadle C. Worledge D. (1999). Daily stem growth patterns in irrigated *Eucalyptus globulus* and *E. nitens* in relation to climate. *Trees* 14 102–111. 10.1007/PL00009752

[B14] Du Z. Cai X. Bao W. Chen H. Pan H. Wang X. (2016). Short-term vs. long-term effects of understory removal on nitrogen and mobile carbohydrates in overstory trees. *Forests* 7:67 10.3390/f7030067

[B15] Elliott G. P. (2011). Influences of 20th century warming at the upper treeline contingent on local-scale interactions: evidence from a latitudinal gradient in the Rocky Mountains, USA. *Glob. Ecol. Biogeogr.* 20 46–57. 10.1111/j.1466-8238.2010.00588.x

[B16] Elliott K. J. Vose J. M. Knoepp J. D. Climton B. D. Kloeppel B. D. (2015). Functional role of the herbaceous layer in Eastern Deciduous forest ecosystems. *Ecosystems* 18 221–236. 10.1007/s10021-014-9825-x

[B17] FAO (2008). *World Reference Base for Soil Resources*. Rome: FAO.

[B18] Grau O. Ninot J. M. Blanco-Moreno J. M. van Logtestijn R. S. P. Cornelissen J. H. C. Callaghan T. V. (2012). Shrub-tree interactions and environmental changes drive treeline dynamics in the Subarctic. *Oikos* 121 1680–1690. 10.1111/j.1600-0706.2011.20032.x

[B19] Gruber A. Wieser G. Oberhuber W. (2009a). Intra-annual dynamics of stem CO_2_ efflux in relation to cambial activity and xylem development in *Pinus cembra*. *Tree Physiol.* 29 641–649. 10.1093/treephys/tpp001 19203979 PMC3013296

[B20] Gruber A. Wieser G. Oberhuber W. (2010). Opinion paper: effects of simulated soil temperature on stem diameter increment of *Pinus cembra* at the alpine timberline: a new approach based on root zone roofing. *Eur. J. For. Res.* 129 141–144. 10.1007/s10342-009-0305-3 21423859 PMC3059491

[B21] Gruber A. Zimmermann J. Wieser G. Oberhuber W. (2009b). Effects of climate variables on intra-annual stem radial increment in *Pinus cembra* (L.) along the alpine treeline ecotone. *Ann. For. Sci.* 66:503. 21423861 10.1051/forest/2009038 PMC3059571

[B22] Hagedorn F. Martin M. Rixen C. Rusch S. Bebi P. Zürcher A. (2010). Short-term responses of ecosystem carbon fluxes to experimental soil warming at the Swiss alpine treeline. *Biogeochemistry* 97 7–19. 10.1007/s10533-009-9297-9

[B23] Hoch G. (2013). Reciprocal root-shoot cooling and soil fertilization effects on the seasonal growth of two treeline conifer species. *Plant Ecol. Divers.* 6 21–30. 10.1080/17550874.2011.643324

[B24] Hoch G. Körner C. (2003). The carbon charging of pines at the climatic treeline: a global comparison. *Oecologia* 135 10–21. 10.1007/s00442-002-1154-7 12647099

[B25] Holtmeier F.-K. (2003). *Mountain timberlines. Ecology, Patchiness, and Dynamics. Advances in Global Change Research* Vol. 14. Dordrecht: Kluwer Academic Publishers 10.1007/978-94-015-1254-1

[B26] Holtmeier F.-K. Broll G. (2007). Treeline advance – driving processes and adverse factors. *Landsc. Online* 1 1–33. 10.3097/LO.200701

[B27] Jarvis P. Linder S. D. (2000). Constraints to growth of boreal forests. *Nature* 405 904–905. 10.1038/35016154 10879523

[B28] Kilian W. Müller F. (1994). Neue Wuchsgebietsgliederung –Folgen für Herkunftsbezeichnung forstlichen Vermehrungsgutes. *Österreichische Forstzeitung* 4 13–15.

[B29] Körner C. (1998). A re-asessment of high elevation treeline positions and their explanation. *Oecologia* 115 445–459. 10.1007/s004420050540 28308263

[B30] Körner C. (2006). “Significance of temperature in plant life,” in *Plant Growth and Climate Change* eds Morison J. I. L. Morecroft M. D. (Oxford: Blackwell Publishing Ltd) 48–69. 10.1002/9780470988695.ch3

[B31] Körner C. (2012). *Alpine Treelines. Functional Ecology of the Global High Elevation Tree Limits.* Basel: Springer 10.1007/978-3-0348-0396-0

[B32] Körner C. Hoch G. (2006). A test of treeline theory on a montane permafrost island. *Arctic Antarct. Alp. Res.* 38 113–119. 10.1657/1523-0430(2006)038[0113:ATOTTO]2.0.CO;2

[B33] Körner C. Paulsen J. (2004). A world-widestudy of high altitide treeline temperatures. *J. Biogeogr.* 31 713–732. 10.1111/j.1365-2699.2003.01043.x

[B34] Kronfuss H. (1997). *Das Klima einer Hochlagenaufforstung in der subalpinen Höhenstufe - Haggen im Sellraintal bei St. Siegmund, Tirol (Periode 1975–1994n).* Vienna: Forstliche Bundesversuchsanstalt.

[B35] Kronfuss H. Havranek W. M. (1999). Effects of elevation and wind on the growth of *Pinus cembra* L. In a subalpine afforestation. *Phyton* 39 99–106.

[B36] Kutschera L. Lichtenegger E. (2002). *Wurzelatlas mitteleuropäischer Waldbäume und Sträucher.* Graz: Leopold Stocker Verlag.

[B37] LeBauer D. S. Treseder K. K. (2008). Nitrogen limitation of net primary productivity in terrestrial ecosystems is globally distributed. *Ecology* 89 371–379. 10.1890/06-2057.1 18409427

[B38] Liang E. Wang Y. Piao S. Lu X. Camarero J. J. Zhu H. (2016). Species interactions slow warming-induced upward shifts of treelines on the Tibetan Plateau. *Proc. Natl. Acad. Sci. U.S.A.* 113 4380–4385. 10.1073/pnas.1520582113 27044083 PMC4843427

[B39] Loranger H. Zotz G. Bader M. Y. (2016). Early establishment of trees at the alpine treeline: idiosyncratic species responses to temperature-moisture interactions. *AoB Plants* 8:plw053. 10.1093/aobpla/plw053 27402618 PMC4988811

[B40] Loris K. (1981). Dickenwachstum von Zirbe, Fichte und Lärche an der alpinen Waldgrenze/Patscherkofel. Ergebnisse der Dendrometermessungen 1976/79. *Mitt. Forstl. Bundesver.* 142 417–441.

[B41] Lupi C. Morin H. Deslauries A. Rossi S. (2012). Xylogenesis in black spruce: does soil temperature matter? *Tree Physiol.* 32 74–82. 10.1093/treephys/tpr132 22210529

[B42] Matsushima M. Chang S. X. (2006). Vector analysis of understory competition, N fertilization, and litter layer removal effects on white spruce growth and nutrition in a 13-year-old plantation. *For. Ecol. Manage.* 236 332–341. 10.1016/j.foreco.2006.09.018

[B43] Melillo J. M. Steudler P. A. Aber J. D. Newkirk K. Lux H. Bowles F. P. (2002). Soil warming and carbon-cycle feedbacks to the climate system. *Science* 298 2173–2176. 10.1126/science.1074153 12481133

[B44] Melillo L. M. Butler S. Johnson J. Mohan J. Steudler P. Lux H. (2011). Soil warming, carbon-nitrogen interactions, and forest carbon budgets. *Proc. Natl. Acad. Sci. U.S.A* 108 9508–9512. 10.1073/pnas.1018189108 21606374 PMC3111267

[B45] Nadelhoffer J. K. Giblin A. E. Shaver G. R. Laiúndre J. A. (1991). Effects of temperature and substrate quality on element mineralization in six Arctic soils. *Ecology* 72 242–253. 10.2307/1938918

[B46] Neuwinger I. (1972). Standortuntersuchungen am Sonnberg im Sellrainer Obertal, Tirol. *Mitt. Forstl. Bundesvers.* 96 177–207.

[B47] Neuwinger I. Wieser G. Winklehner W. Heiss G. (1988). Soil water investigations at a high-level afforestation area near Haggen in the Sellrain Valley, Tirol. *Österreichische Wasserwirtschaft* 40 57–61.

[B48] Nilsson M. C. Wardle D. A. (2005). Understory vegetation as a forest ecosystem driver: evidence from a northern Swedish boreal forest. *Front. Ecol. Environ.* 3:421–428. 10.1890/1540-9295(2005)003[0421:UVAAFE]2.0.CO;2

[B49] Oberhuber W. (2017). Soil water availability and evaporative demand affect seasonal growth dynamics and use of stored water in co-occurring saplings and mature conifers under drought. *Trees* 31 67–478. 10.1007/s00468-016-1468-4 28381902 PMC5375970

[B50] Oberhuber W. Kofler W. Pfeifer K. Seeber A Wieser G. (2008). Long term changes in tree-ring–climate relationships at Mt. Patscherkofel (Tyrol, Austria) since the mid-1980s. *Trees* 22 31–40. 10.1007/s00468-007-0166-7 21532976 PMC3083837

[B51] Okano K. Bert-Hane M. S. (2015). Warming and neighbor removal affect white spruce seedling growth differently above and below treeline. *Springer Plus* 4:79. 10.1186/s40064-015-0833-x 25729635 PMC4339320

[B52] Piper F. I. Vinegla B. Linares J. C. Camarero J. J. Cavieres L. A. Fajardo A. (2016). Mediterranean and temperate treelines are controlled by different environmental drivers. *J. Ecol.* 104 691–702. 10.1111/1365-2745.12555

[B53] Platt K. H. Allen R. B. Coomes D. A. Wiseer S. K. (2004). Mountain beech seedling responses to removal of below-ground competition and fertiliser addition. *N. Z. J. Ecol.* 28 289–293.

[B54] Rainer G. Kuhnert R. Unterholzer M. Dresch P. Gruber A. Peintner U. (2015). Host-specialist dominated ectomycorrhizal communities of *Pinus cembra* are not affected by temperature manipulation. *J. Fungi* 1 55–75. 10.3390/jof1010055 29376899 PMC5770009

[B55] Rossi S. Deslauries A. Anfodillo T. Carraro V. (2007). Evidence of threshold temperatures for xylogenesis in conifers at high altitudes. *Oecologia* 152 1–12. 10.1007/s00442-006-0625-7 17165095

[B56] Rossi S. Deslauries A. Anfodillo T. Morin H. Saracino A. Motta R. (2006). Conifers in cold environments synchronyze maximum growth rate of tree-ring formation with day length. *New Phytol.* 170 301–310. 10.1111/j.1469-8137.2006.01660.x 16608455

[B57] Rossi S. Morin H. Deslauries A. (2011). Multi-scale influence of snowmelt on xylogenesis of black spruce. *Arctic Antarct. Alp. Res.* 43 457–467. 10.1657/1938-4246-43.3.457

[B58] Rustad L. E. Campbell J. L. Marion G. M. Norby R. J. Mitchell M. J. Hartley A. E. (2001). A meta analysis of the response of soil respiration, net nitrogen mineralization, and aboveground plant growth to experimental ecosystem warming. *Oecologia* 126 543–562. 10.1007/s004420000544 28547240

[B59] Smith W. K. Germino M. J. Johnson D. M. Reinhardt K. (2009). The altitude of alpine treeline: a bellwether of climate change. *Bot. Rev.* 75 163–190. 10.1007/s12229-009-9030-3

[B60] Song H. Cheng S. Zhang Y. (2016). The growth of two species of subalpine conifer saplings in response to soil warming and inter-competition in Mt. Gongga on the south-eastern fringe of the Qinghai-Tibetan plateau, China. *World J. Eng. Technol.* 4 398–412. 10.4236/wjet.2016.43039

[B61] Strömgren M. Linder S. (2002). Effects of nutrition and soil warming on stemwood production in a boreal Norway spruce stand. *Glob. Ghange Biol.* 8 1194–1204. 10.1046/j.1365-2486.2002.00546.x

[B62] Susiluoto S. Hilasvuori E. Berninger F. (2010). Testing the growth limitation hypothesis for subarctic Scots pine. *J. Ecol.* 98 1186–1195. 10.1111/j.1365-2745.2010.01684.x

[B63] Sveinbjörnsson B. (2000). North American and European treelines. External forces and internal processes controlling position. *AMBIO* 29 388–395. 10.1579/0044-7447-29.7.388

[B64] Sveinbjörnsson B. Nordell O. Kauhanen H. (1992). Nutrient relations of mountain birch growth at and below the elevational tree-line in Swedish Lapland. *Functional Ecology* 6 213–220. 10.2307/2389757

[B65] Tranquillini W. (1979). *Physiological Ecology of the Alpine Timberline. Tree Existence in High Altitudes with Special Reference to the European Alps.* Berlin: Springer 10.1007/978-3-642-67107-4

[B66] Vaganov E. A. Hughes M. K. Kirdyanov A. V. Schweingruber F. H. Silkin P. P. (1999). Influence of snowfall and melting time on tree growth in subarctic Eurasia. *Nature* 400 149–151. 10.1038/22087

[B67] Walther G.-R. Beißner S. Pott R. (2005). “Climate change and high mountain vegetation shifts,” in *Mountain ecosystems. Studies in Treeline Ecology* eds Broll G. Keplin B. (Berlin: Springer) 77–95.

[B68] Wardle P. (1974). “Alpine timberlines,” in *Arctic and Alpine Environments* eds Ives J. D. Barry R. (London: Methuen Publishing) 371–402.

[B69] Weih M. (2000). Delayed growth response of mountain birch seedlings to a decrease in fertilization and temperature. *Funct. Ecol.* 14 566–572. 10.1046/j.1365-2435.2000.t01-1-00452.x

[B70] Weih M. Karlsson P. S. (2001). Growth response of mountain birch to air and soil temperature: Is increasing leaf-nitrogen content an acclimation to lower air temperature? *New Phytol.* 150 147–155. 10.1046/j.1469-8137.2001.00078.x

[B71] Wieser G. Grams T. E. E. Matyssek R. Oberhuber W. Gruber A. (2015). Soil warming increased whole-tree water use of *Pinus cembra* at the treeline in the Central Tyrolean Alps. *Tree Physiol.* 35 279–288. 10.1093/treephys/tvp009 25737326 PMC4820648

[B72] Wieser G. Matyssek R. Luzian R. Zwerger P. Pindur P. Oberhuber W. (2009). Effects of atmospheric and climate change at the timberline of the Central European Alps. *Ann. For. Sci.* 66:402. 10.1051/forest/2009023 21379395 PMC3047780

[B73] Wieser G. Tausz M. (2007). *Trees at Their Uppert Limiz. Treelife Limitation at the Alpine Timberline.* Dordrecht: Springer.

